# Visibility of documentary heritage through digitisation projects in Romanian libraries

**DOI:** 10.1371/journal.pone.0280671

**Published:** 2023-01-23

**Authors:** Maria Micle, Elena Tîrziman, Angela Repanovici

**Affiliations:** 1 Faculty of Political Sciences, Philosophy and Communication Sciences, West University of Timisoara, Timișoara, Romania; 2 Faculty of Letters, University of Bucharest, Bucharest, Romania; 3 Faculty of Product Design and Environment, Transilvania University of Brasov, Brașov, Romania; King Fahd University of Petroleum & Minerals, SAUDI ARABIA

## Abstract

**Background:**

The article presents, from a diachronic perspective, the concerns of valorization and preservation of the documentary heritage through digitisation projects in the Romanian Library System. The development of the argument is made starting from the analysis of the interest in the subject in international literature (through a bibliometric analysis) and the investigation of the online presence of libraries expressed statistically from the typology of forms of representation, to specific digital products and services, thus highlighting how they evolved in the sense of diversifying and modernizing the products and services provided and taking into account the political, economic, and technological constraints.

**Methods:**

The evaluation of the involvement of Romanian libraries in digitisation activities was done through the such research methods as content analysis and scientometric research, which allowed the carrying out of a longitudinal case study extended for the period 2007–2022, during the unravelling of the events.

**Results:**

The most representative initiatives for the creation of the Digital Library of Romania and the involvement of libraries in national and European projects are presented, and examples of good practices are highlighted, including political and administrative concerns to support protection and capitalization through the digitisation of the libraries’ heritage. This study is the first in Romanian specialized bibliography that proovides an overall and evolutionary perspective on the national project of digitisation of the documentary cultural heritage. Analyzing a large number of official documents and library web addresses has been a major challenge.

**Conclusions:**

During the analyzed period (2007–2022), there have been numerous initiatives and digitisation projects, but the results have not always met expectations. From the experience of Romanian libraries, it can be concluded that digital content development initiatives have not always been successful because of the absence or insufficient commitment of the sustainability component.

## Introduction. The research context

The last two years have caused multiple changes at social, political, economic, and environmental levels. The crisis caused by the COVID-19 pandemic in the last two years has been a real challenge for libraries and other information and documentation structures: they have been put in a position to serve their specific users remotely through digital products and services and less through products and services traditionally provided in the physical space of institutions. Although it is a real crisis situation, the pandemic was, for libraries, an opportunity to realistically analyse their place and role in society, the ability to provide real distance access to accurate filtered, trustworthy or intermediate information resources, to provide competences and institutional resources needed to create its own digital content starting from their own collections (mainly heritage collections). Romanian libraries have also faced the need to transfer in the online environment most activities and to increase the volume of digital products and services provided to users.

The goal of this study was to identify and describe the steps made at national level to digitise the libraries’ written documentary heritage and to create the Digital Library of Romania as an evolutionary approach and as part of the collective effort of libraries to capitalize by digitisation. The monitoring period of this case study was 2007–2022, that is, from the beginnings of projects of this type to their current stage.

Specific objectives derived from this general objective are related to the identification of existing initiatives; good practices regarding the digitisation of documents; collections of representative Romanian libraries to promote and capitalize on the national and local representative heritage; products and services in digital format to meet users’ information needs; and contribution to the improvement of the online presence of libraries, both in terms of informational offer and, implicitly, of image. All these objectives are pursued as component parts of an evolutionary process of establishing the Digital Library of Romania–a large-scale national emergency project. In order to clarify the cultural and administrative context in Romanian libraries, it is worth mentioning that the national library system consists of the following categories of libraries: the National Library [NLR], public libraries, libraries in the education system, and specialized libraries. At the administrative level, they are subordinated to two ministries: the Ministry of Education (those in academic and pre-academic education) and the Ministry of Culture (the National Library, public libraries), as well as subordinated to local authorities such as county councils or mayoralties (county, municipal, city, and communal public libraries). The professional community is represented by two professional associations: the Romanian Libraries Association and the National Association of Public Libraries in Romania [1].

Statistical data on the online presence of Romanian libraries shows, synthetically, how they have evolved over the last thirty years in the understanding of their activities, diversification and modernization of products and services provided to users while taking into account the constraints of political, economic, and technological factors. The data transposed graphically in Fig 2 were collected from the direct query of the CASIDRO [Catalogue of Info-Documentation Structures in Romania] [2] and through direct analysis and comparison with the web pages of the libraries (county ~, university ~, etc.). CASIDRO is a portal including currently over 90% of Romania’s libraries of all types. It covers descriptive information about each library and it is the result of a project developed by the National Library concomittantly with the materialisation of the policy of digitisation of documentary funds. The platform functions based on a unique access point of each participant library and is an important source of information for the academic and librarian communities when they need to identify Romanian libraries. This referrential is updated on a continuous basis and has been an important common source of documenting for the materialisation of the digitisation of collections and of a unique digital library in Romania. This model can be replicated and used in other national projects as well.

Considering the online institutional presence as any form of institutional representation that contributes to the knowledge and promotion of the library, both in the community it serves and in the current informational environment, we have taken into account libraries, blogs, presentation pages, profiles in social media, digital products and services developed by libraries or for which they provide intermediary access (online catalogues, digital libraries, digital collections, electronic resources access, and discovery services).

**Fig 1:**
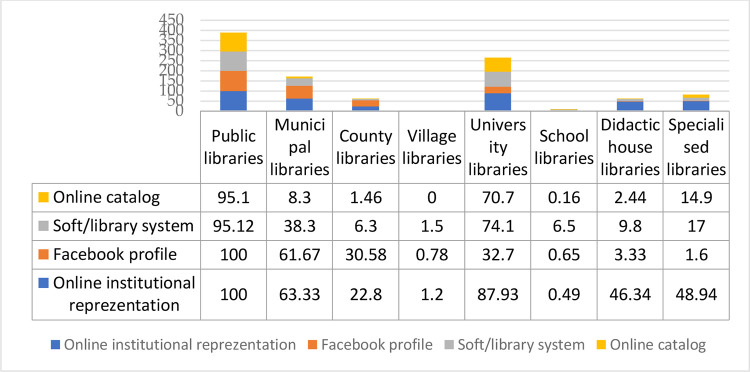
Synthetic representation of the types of libraries in Romania and their online presence: Facebook, specialized collection and user management software, online catalogue (CASIDRO data [2]).

At the beginning of 2022, 3642 libraries were recorded in the CASIDRO database: the National Library of Romania [NLR], the Romanian Academy Library, 41 county libraries (including the Bucharest Metropolitan Library), 60 municipal libraries, 206 city libraries, 2569 communal libraries, 58 university libraries, 47 specialized libraries, 618 school libraries and information and documentation centres, and 41 libraries of the Teaching Staff Houses representing about 90% of the Romanian libraries [2] (Fig 1). The presence of the online library can be made in the following ways: institutional website, institutional blog, page within the website of the patronage authority (local authority, university, high school, school, institute, etc.), social media [3].

Unfortunately, although the number of libraries is relatively high, when analysing their actual presence in the online environment, around 255 of them have their own website, that is a web address (large libraries). Small libraries are present online as a page in the website of patronage institutions in the form of blog or through social networks. Therefore, when talking about the development of digital content and of publishing on the site, of protecting and capitalizing through digitisation the documentary heritage of libraries, one needs considering large libraries that have an institutional site.

Identifying the different approaches to digitisation in Romanian libraries allows a general assessment of the digitisation at national level of library documents in terms of projects, good practices, forms of collaboration and sharing of activities, products and services, definition premises and principles for the development of the Digital Library of Romania.

## Materials and methods

To examine the current state of the online institutional presence of libraries and of the digitisation projects in Romania, the authors have used **a mix of research methods [4], quantitative and qualitative ones, to achieve a longitudinal case study [5] over the period 2007–2022**, pursuing and collecting data on the characteristics of the national library system. Professional immersion and authors’ expertise in the Romanian librarianship area allowed them to directly observe evolution and get an overall perspective on the subject of the study.

The methods used to give coherence and critical reflection on the topic for data collection and analysis are: content analysis, scientometric research, the correlational model of keyword clusters, and case study.

The content analysis method. For the collection and presentation of data on the visibility of Romanian libraries in the online environment, the online catalogue CASIDRO–Catalogue of Info-documentary Structures in Romania was used, an open access resource managed by the NLR and developed collaboratively by having each library listed in this catalogue provide its own information. Data was collected by punctually querying the CASIDRO catalogue, which resulted in the total number of libraries and their distribution by type. Each library’s listing contains information about its online presence through the web pages of the libraries or institutions they serve or through blogs. Results obtained in graphical form in Figs 3 and 4 is also presented. For the collection of data on initiatives, digitisation projects and the presence of libraries, the Manuscriptorum platform [29] was used, as well as the direct analysis of the web addresses of the mentioned libraries. The selection of libraries was based on the criterion of relevance (the number of documents included in the mentioned platform).

Data for the case study was completed by identifying and selecting the official public primary documents supporting and capturing the whole process of genesis and becoming the process of establishing the Digital Library of Romania, administratively, politically (involving decision-making fora–Ministry of Culture and Cults, the European Commission, the National Library) which were capitalized on in *Digitisation Initiatives at the Level of Romanian Libraries* below.

The methods used to give coherence and critical reflection on the subject for data collection and analysis are content analysis [6], scientometric research, correlational model of key word clusters, and case study–as a predominant research strategy.

### Scientometric analyses

The research methodology used to obtain the results synthesized in Part 3 (*Results*) is preceded and complemented by a scientometric content analysis to pursue, as a premise of the topicality of the study, the relevance of the chosen theme, the reflection of interest and application for the topic investigated in literature. Scientometrics deals with the measurement and analysis of a particular scientific field [7]. Currently, bibliometric research methods are of interest to the international scientific community, especially in the higher education area because it allows the analysis of a significant flow of documents to assess the performance of research or for the analysis of the reading / study interests of the readers [8] (p. 265). Using bibliometric research, scientometrics analyses quantitative characteristics and scientific research in the selected field. In this research, the VOS Viewer software, which provides advanced viewing tools for building and visualizing bibliometric networks, has been used. These networks may include, for example, magazines, research or individual publications and can be built on citation, bibliographic coupling, co-citation, co-author relationships, or geographic influences on the researched field. As regards the choice of the database, for reviewing specialist publications, the “Web of Science” database was chosen as one of the most notorious at international level. The search was done using the search phrase *digitisation* and *heritage* and *libraries*, for which 215 results were obtained. Subsequent refining of these results by including the information criterion in the field of Information science, library science generated 110 results. Analysing the typology of the 110 documents, it was clear that 67 were articles (Table 1).

**Table 1:** Scientometric study stages.

Steps	Description
Formulation of the problem	Mapping, bibliometric analysis of publications using descriptors, and research direction identification.
Research criteria	Subject:"(ALL = digitization AND heritage AND libraries)
Database used for research	Claryvate analytics, WEB OF SCIENCE—WOS
	Accessed on
Eligibility criteria	Filter 1: years of publication (2007–2022)
	Result: 215 documents.
	Filter 2: articles
	Filter 3: English
	Filter 4: Open access 110 documents
	Result: 67 documents.
Data extraction	Bilingual format
Analysis and synthesis of results	Qualitative (descriptive) and quantitative (bibliometric) using VOS Viewer
Discussions	Analysis of the data gained

**Fig 2:**
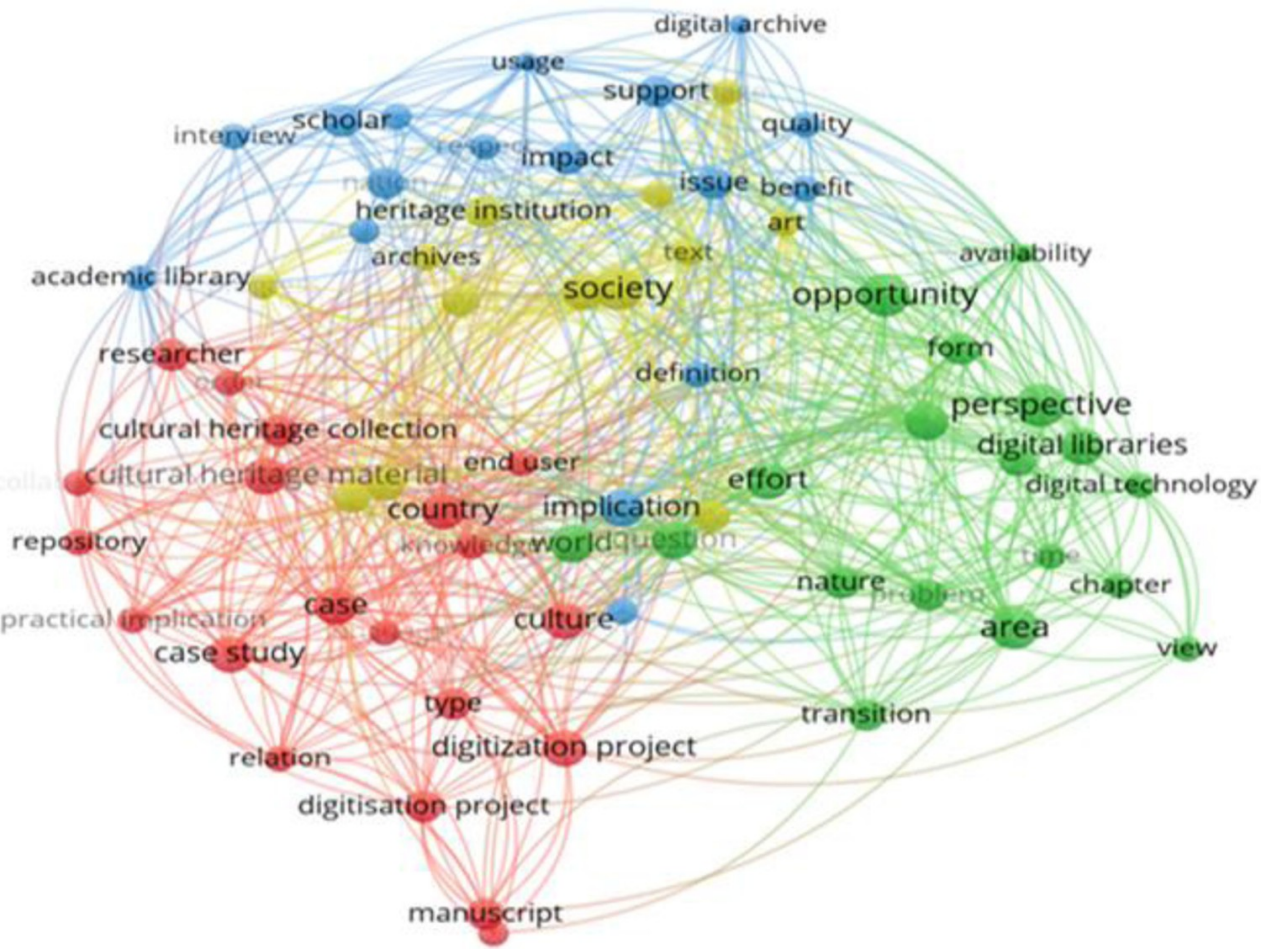
Keyword visualization map.

The plaintext database from Web of Science was downloaded and the words appearing in the title and summary were analysed. The condition was imposed that a word to appear at least 3 times. Of the 1404 words, 113 met the condition. Using the VOS Viewer software, the word visualization map was generated (Fig 2). Four clusters were identified: the red one identifies the direction of digitisation through cultural heritage collections, the green one, the direction of the perspectives of digital libraries and digital technology, the blue one, the direction of education, impact and academic libraries, and the yellow one, societies and heritage protection institutions.

**Fig 3:**
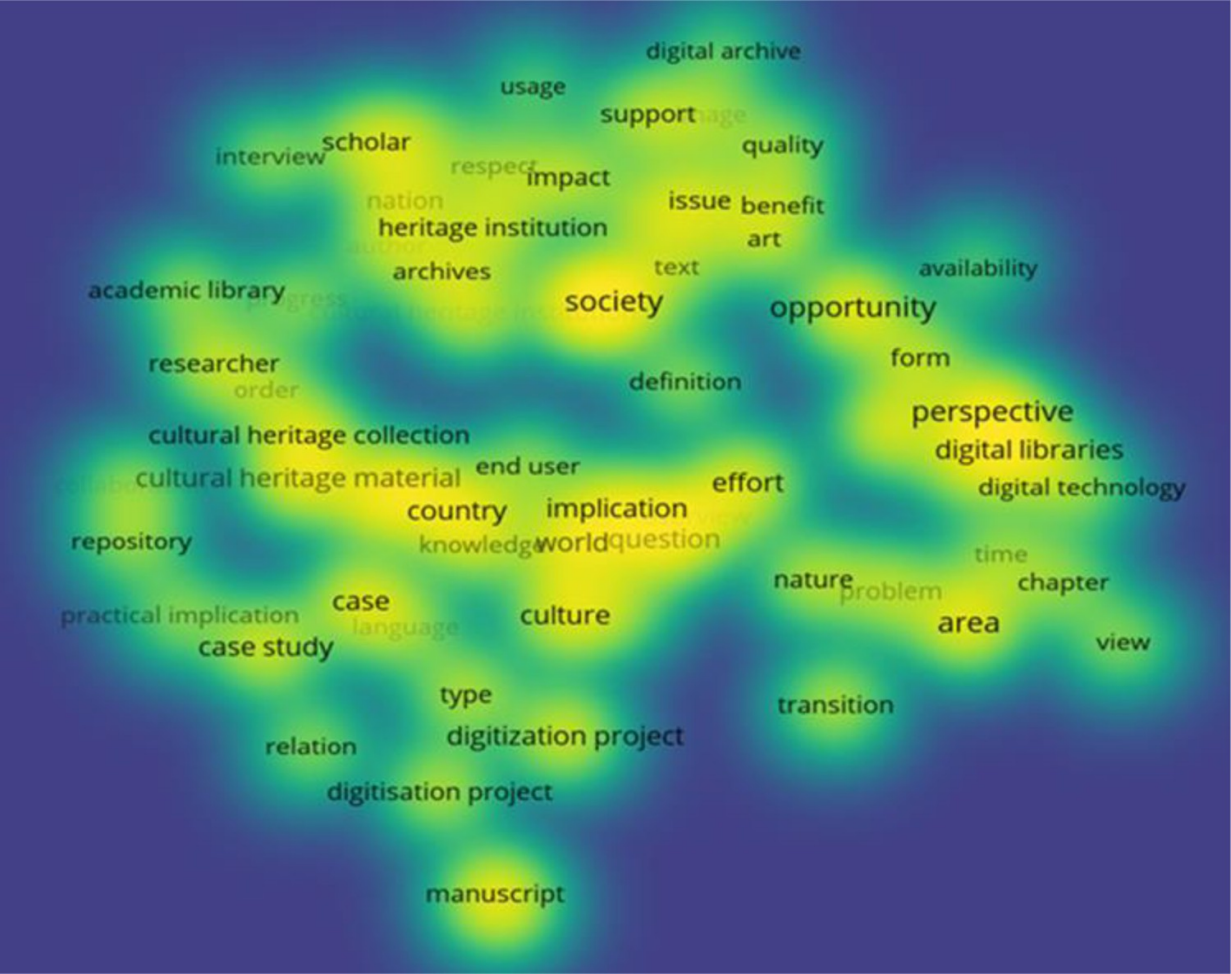
Word density map used in title and summary.

In Fig 3, the density map was chosen as a visualization method. Yellow areas present words with the greatest impact in general research.

Scientometric analysis indicates the international interest in the topic discussed and in subjects such as material cultural heritage, perspectives on digital library, and organization of knowledge through digital projects and deposits.

### Case study

The case study was carried out in order to extract the characteristics of the Romanian system of libraries in the digitisation area for the protection and dissemination of the documentary heritage and, thus, to find out how the situation is interpreted in the Romanian area.

The case study was chosen as a relevant research method because it is widely used in social sciences in treating themes in contemporary areas seen in the context of real life, with practical or public policy assessment [5] as is this research subject: the draft Documentary Heritage of Romanian Libraries, seen as an evolutionary, open process that pursues TRSEUL for the establishment and application of digitization policies for accessibility of collections. The evolution analysis of the process provides the opportunity to share expenses with other libraries in the world.

The method of the descriptive case study allowed the extraction of the characteristics of setting up and developing the digitisation projects in the succession of events and in their organizational context. In using the case study as a method, the professional experience of authors in the library field is paramount such as that of co-author Elena Tîrziman. She was the director of the NLR during 2007–2014 and was directly and intensely involved in the definition and implementation of the digitisation policies in Romania.

The empiric material selected comprised all official documents that have founded the policies and digitisation projects of written cultural heritage; they were analysed to reconstruct and provide an overall perspective on the entire digital heritage. Vast Romanian digitisation projects were also selected, which are presented as models of good practices.

## Results

Technology has revolutionized people’s way of life giving them opportunities for accessing the humanity’s heritage of knowledge unimaginable a few decades ago: it is the time of transition to virtual reality. The new communication behaviours of society in the virtual space bring about tremendous benefits, such as the democratization of knowledge. It also brought about anomalies: unprecedented propagation of misinformation against which libraries bring remedies by expanding digital deposits and collections. By accessing as diverse digital collections as possible, the effects of misinformation decrease because it removes information in the area of non-verification. Print documents set accessibility limits and one can consider that it handcuffs the information in their material support, limiting their circulation; or, at present, the accessibility and dissemination of information in the virtual space allow easy collection of evidence, especially those used in scientific communication. Digital collections, transmedia resources, therefore, provide and bring out the evidence that science needs to progress and persuade. “Ever since the idea of the library as an instrument of education and a place of knowledge has been considered the essential mission of the modern library. When knowledge was predominantly encoded in printed text resources, libraries were promoting skills and providing access to books and journals. With new media forms such films, video games or digital apps rising to social and cultural prominence, libraries started to build audio-visual collections, carry out digitisation projects and introduce new technology services” [9] (p. 4).

The information society has been organized both as a system and as a network, therefore digitisation projects, in order to achieve multiple functionalities, need coordination from the relevant ministries, the collaboration of the professional environment, and the support of public authorities in sharing resources with users. Libraries are involved in the circulation of information, and digital collections and services are an adaptation to the current knowledge society, fluid, digitisation-oriented and to the virtual manifestation area.

**Fig 4:**
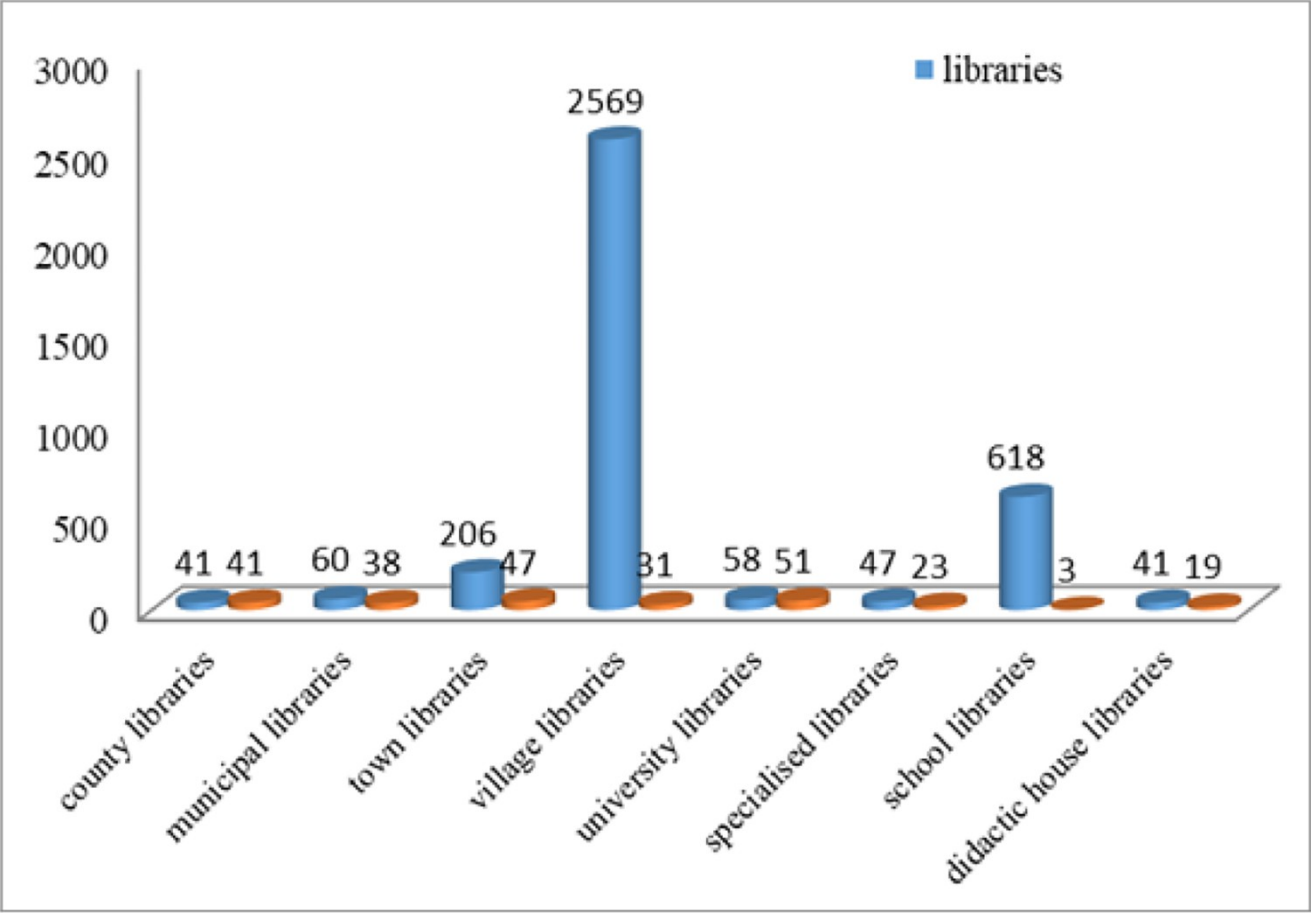
Libraries in the online environment, reported to the total number of libraries, by types.

Regarding the network of Romanian libraries and their accessibility in virtual space to help people know more about their services and who would otherwise not visit them in person, one can see that the percentages of their institutional presence in the online environment vary greatly from one type to another. The best represented are county libraries (100%), followed by university libraries (87.93%) and municipal libraries (63.33%). Less than half of specialized libraries (48.94%), teaching staff libraries (46.34%) and city libraries (22.82%) are active in the online environment. The least represented are school libraries (0.49%) and communal libraries (1.20%): all large and medium-size libraries of the country are registered, but all school libraries or all small libraries of institutions are not yet registered [3] (Fig 4).

**Fig 5:**
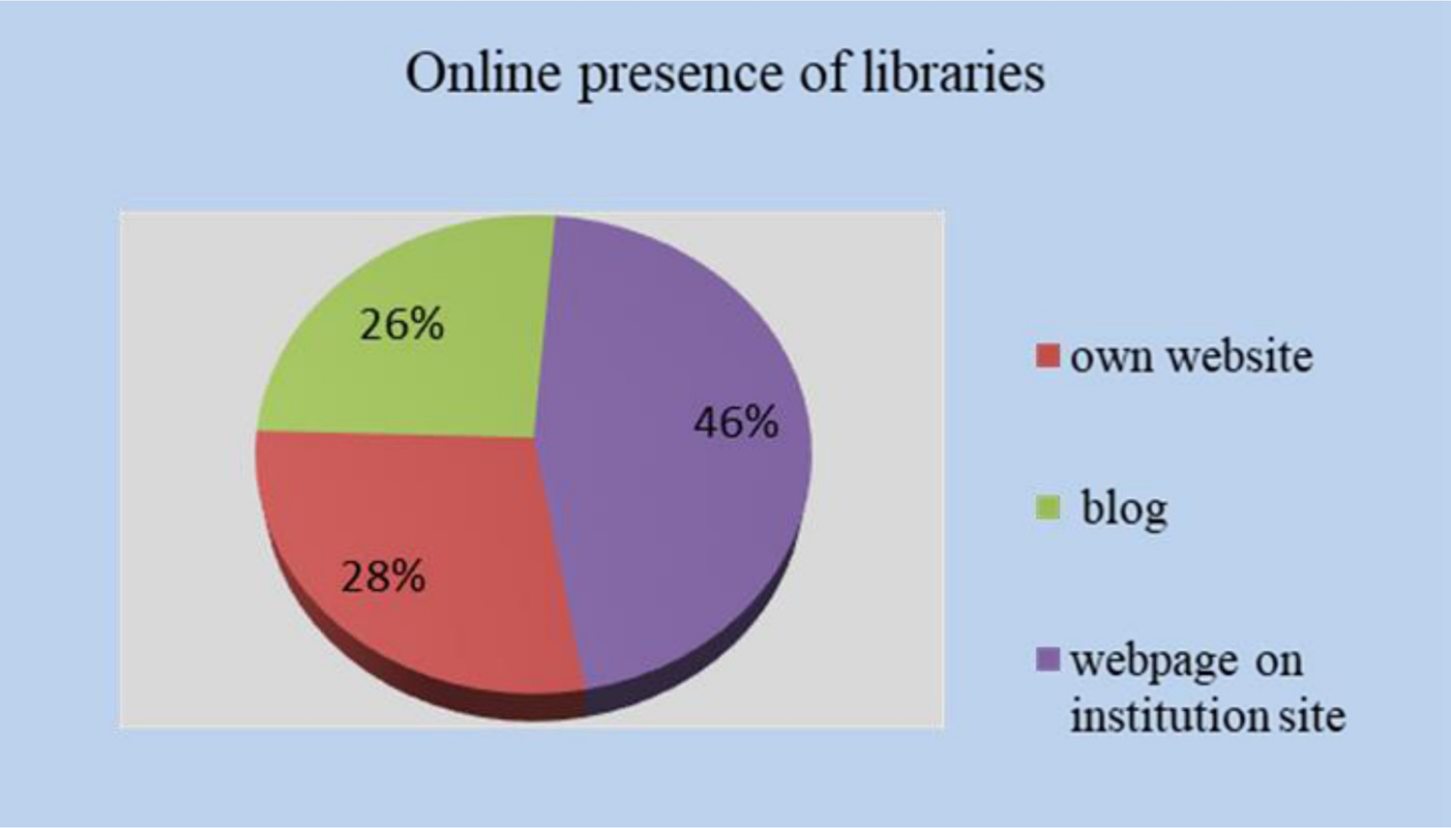
Online presence of Romanian libraries.

From the perspective of online presence, 72 libraries have an institutional website, 66 libraries are represented by a blog, and 117 libraries are described (sometimes, just mentioned) on a page or in a section of the patronage institution’s website (Fig 5).

Regarding the digitisation of their own collections in Romania, digitisation activities are considerable, but efforts in this direction are uneven, sometimes disparate, and the rhythm is not the expected one. The causes are, in most cases, financial, organizational, and decision-making. Institutional initiatives resulting from the desire to publish representative documents from collections or documents due to involvement in projects aimed at creating and publishing digital collections and digitising for protection are notable.

Along with institutional initiatives, to also note the political, administrative concerns for supporting the entire Romanian National Libraries System in the attempts to protect and capitalize documentary cultural heritage through digitisation. Thus, when talking about digitisation at the level of the national library system, on needs to take into account both the political-administrative approach of recovery by digitisation of documentary heritage, and the individual or collaborative achievements of Romanian libraries.

### Initiative for the achievement of the Romanian Digital Library

A political-administrative approach to the digitisation of documentary heritage at national level has been achieved as a result of the need to adopt at national level the European Commission’s recommendations 2006/585/EC and 2006/C 297/01 [10, 11]. The Ministry of Culture and Cults had the initiative to promote a Public Policy for achieving Romania’s Digital Library in line with the two recommendations of the European Commission. The NLR was involved in defining and substantiating this public policy. All procedural steps for defining, promoting, and supporting the Public Policy Proposal were carried out between August and December 2007. On 18 January 2008, this Public Policy was approved by the Romanian Government and could be consulted on the Romanian Ministry of Culture website [12].

The principle they started from in defining this public policy was that the Digital Library of Romania is a unitary structure of representation of digital national cultural heritage, while both observing the accessibility to digital resources through a single access point and observing a thematic and institutional organization. Thus, the following themes were defined: Written documentary heritage (libraries); Mobile heritage (museums, collections); Audio-video heritage (audio-video archives); Immobile heritage (monuments, archaeology); Archival heritage (at that time, it was decided that it be addressed at a later stage, being the subject of another public policy document).

For the thematic module or pillar libraries, the NLR started with the initiation of a feasibility study in the public library system in Romania to identify the minimum conditions for promoting and implementing such a public policy in the field of libraries [13]. This study consisted in an institutional survey based on a questionnaire initiated and coordinated by Professor Elena Tîrziman, distributed in 2007 to the following libraries: county libraries (which also have a methodological role within the territory served), the Library of the Romanian Academy, and the “Carol I” Central University Library in Bucharest. Subsequently, the prospectus was expanded, and the questionnaire was distributed to other university libraries to extend research results. The aspects of information and documentary resources held by libraries, their involvement in digitisation activities and projects, human and technological resources, possible difficulties, and the vision of digitisation of collections were pursued. The analysis of the data supplied by the libraries investigated at the time suggested a few ideas reflecting the reality, which led, in parallel, to the initiative of the National Library of developing CASIDRO as a complementary instrument in the digitisation process:

- Libraries have funds on material support structured according to the typology of documents: books, periodicals, manuscripts, cartographic documents, audio-video documents, and graphic documents. The size of the collections varies depending on the type of library and, at local level, on the approved budget.
- A number of individual small-scale digitisation projects were carried out within the libraries. In most cases, digitised documents are provided for user consultation, locally or through library web pages.
- All libraries have both traditional (alphabetical, systematic, etc.) and electronic catalogues, most using specialized library software.
- In the case of public libraries, purchased electronic resources are scarce (Romanian legislation database, Oxford Journals, EBSCO, etc.), but such resources are found with greater diversity in university libraries.
- IT infrastructure at library level varies depending on the budget of each library and institutional management. Most county libraries use Tinlib as integrated library software, but there is also Aleph, Alephino, Qulto, Bibliophil softwares.
- There are a small number of IT specialists in libraries, which has a positive impact on the smooth deployment of the national digitisation project.
- Libraries propose for digitisation a selection of periodical publications from the local press, the “Monitorul Oficial” collection, as well as Romanian old books or school bibliography.

The main themes pursued in the feasibility study elaborated by the NLR were the national legislative framework, the objectives envisaged by libraries in the digital transposition of documentary heritage, the information and documentation resources in Romanian libraries, and the way in which they are preserved and capitalized to generate an approach and a unitary working methodology at national level.

The national digitisation strategy aimed at identifying existing digitisation projects, identifying digitisation priorities of libraries and documents proposed for digitisation, selecting the documents / collections to be digitized, defining criteria for establishing the representative documentary corpus (legal aspects with their two components–intellectual property rights and broadcasting rights), identifying equipment and software, digitisation solutions, training people in the library, and calculating the costs involved. Specific information was reported and many were updated through the Digital Library Compartment in the Romanian National Library.

Much of the problems presented in the feasibility study conducted by the NLR were taken over in the Public Policy of the Ministry of Culture on digitising national cultural resources but, unfortunately, no funding was approved for such a national program for digitisation of the written cultural heritage. The initiative for achieving the Romanian Digital Library has remained a priority objective and has been included among the priorities of the Ministry of Culture.

The year 2014, when Romania’s Digital Library should have been completed and connected to the European digital library, was actually the re-launching of this vast national project. The European Commission, through the 2014–2020 Competitiveness Operational Program (AM POC), EC Decision C(2014)10233-19.12.2014 [14] included clarifications on the development of digital content in the field of culture. The preparation of the project was carried out during 2016–2018: discussions and negotiations were held for the implementation and finalization of the Applicant’s Guide by the Ministry of Communications and Information Society, and the Ministry of Culture and National Identity carried out specialized studies (a second feasibility study conducted within a decade–and the technical project) necessary for the submission of the project. At the end of 2018, it was applied for funding. The Digital Library of Romania, called E-Culturalia Project, received a funding of 11.4 million euros. It took a 12-year period (2007–2019) for the legal, economic and administrative framework to achieve the National Digitisation Program with its most important project, the Digital Library of Romania (with a public policy in the digital field and the necessary financial allocation) to be completed. E-Culturalia has two major objectives: achieving the culturalia.ro platform and the digitisation of the national mobile cultural heritage, i.e., digitisation, description and display, in the Digital Library of Romania, of 550,000 cultural resources from which about 200,000 cultural resources to be digitized and supplied to europeana.eu by 2021.

Twenty-nine public institutions are involved in this project, among which the Metropolitan Library in Bucharest, the Astra Sibiu County Library, the “Octavian Goga” County Library in Cluj-Napoca, the “C. Sturdza” County Library in Bacau, the “Mihai Eminescu” Central University Library in Iasi, the National Film Archive, the Romanian Television Society, the Romanian Broadcasting Society, and the National Heritage Institute (the last four, in Bucharest).

### Basic principles in achieving the digital library of Romania

Relations established between the libraries in the National Library System in Romania, formal or informal, are determined by the attributions and limits on the digitisation of documentary heritage at national level. The main difficulty in the relationship between libraries is the legal functioning framework. Thus, the National Library is subordinated to the Ministry of Culture, which is a central authority; the Romanian Academy Library is subordinated to the Romanian Academy, which is a structure in the field of private heritage of the state; county libraries are subordinated to the county councils, municipality, city, and communal libraries are subordinated to mayoralties, i.e., to local authorities; central university libraries are subordinated to the Ministry of Education; other university libraries, as well as school libraries, are integrated in the institution they serve without legal personality and, therefore, with a limited decision-making power. Such administrative and budgetary heterogeneity cannot be without consequences in the unitary operation of libraries at the level of the National Library System. The trans-institutional aspects bringing together Romanian libraries in the National Library System are unitary librarianship practice, unitary norms and rules, existence (at least, theoretical) of methodological functions, integration of libraries and librarians in specific networks and associations [15].

Taking into account the concrete situation of Romanian libraries from the point of view of the administrative subordination, the librarians’ used practices and relationship through professional associations, the achievement of a national digital library is only possible by observing principles for the creation, development and use of this complex informational product, of hoarding up heritage, and also of information and documentation service. The principles for its achievement are:

1. The principle of national coordination of activities for the purpose of unitarily addressing the processes involved, ensuring communication with the institutions involved, promoting and supporting the legal, administrative, financial, and professional framework, evaluating the results, and correcting the errors.
2. The principle of sharing, similar to the Shared National Catalogue, in order to efficiently use the resources involved, to avoid duplication of digitisation activities for the same documents, and to create the premises for specialization in certain services or activities.
3. The principle of using norms, unitary standards to level the working methods, ensuring the compatibility of the IT systems involved, and presenting digitized documents and collections in a unitary form.
4. The principle of unitary planning of activities short-term (1 year) or medium-term (4–5 years) to establish the representative documentary corpus, to set priorities in digitisation, to choose digitisation solutions, and to estimate the cost involved.
5. The principle of interoperability in establishing communication through IT specific protocols systems that provide communication infrastructure so that libraries can contribute to collections and digital library with other institutions and to ensure the promotion, integration of Romanian heritage in European and world heritage; the National Digital Library should connect nationally dispersed databases and integrate them into a unitary national system.
6. The principle of integration to recover the results of previous projects and their integration into a unitary system within the Digital Library of Romania, as well as the integration of digitisation results from different institutions into a unitary interface.
7. The principle of proper legal framework to ensure compliance with intellectual property rights at all stages of the project and in all aspects involved.
8. The principle of sustainability to permanently ensure the financial, technological, information, and human resources involved in the development and exploitation of the Digital Library of Romania [16, 17].

### Digitisation initiatives at the level of Romanian libraries

A consequence of the approval of the Public Policy of achieving Romania’s Digital Library was the establishment of a specialized Commission for libraries with the role of analysing reality on computerization and digitisation in the Romanian library system.

The library specialized commission gathered during the years 2008–2010 in workshops and succeeded in making a comprehensive inventory of what was already digitized in Romanian libraries, an estimate of what was intended to be a priority, an estimate of hardware and software needs, a proposal for the mode of representation in the Internet of cultural resources, an identification of copyright and related rights for documents held by libraries [18].

The NLR initiated an Inventory list [19] that signals digitized documents by libraries in the national library system. Without the claim to be exhaustive, this list can be a referential on what to digitize in libraries and collaborative actions; it could help avoid duplication of digitisation of the same documents by several institutions and, thus, harmonize the resources involved and avoid unnecessary or additional costs. In 2009, the methodology for updating the Inventory list that was communicated to all libraries in the National Library System. These forms were published on the National Library website and, on their basis, information on the emergence of new digitized documents is collected and centralized and updates of the inventory list are made. Unfortunately, libraries did not complete these forms and were not concerned about updating their information on their digitisation activities. Consequently, libraries do not know the activity of other similar digitisation institutions, so there are situations of doubling or multiplying scanned and digitized document, of cost increases, of ignoring specific good practices, etc.

**Fig 6:**
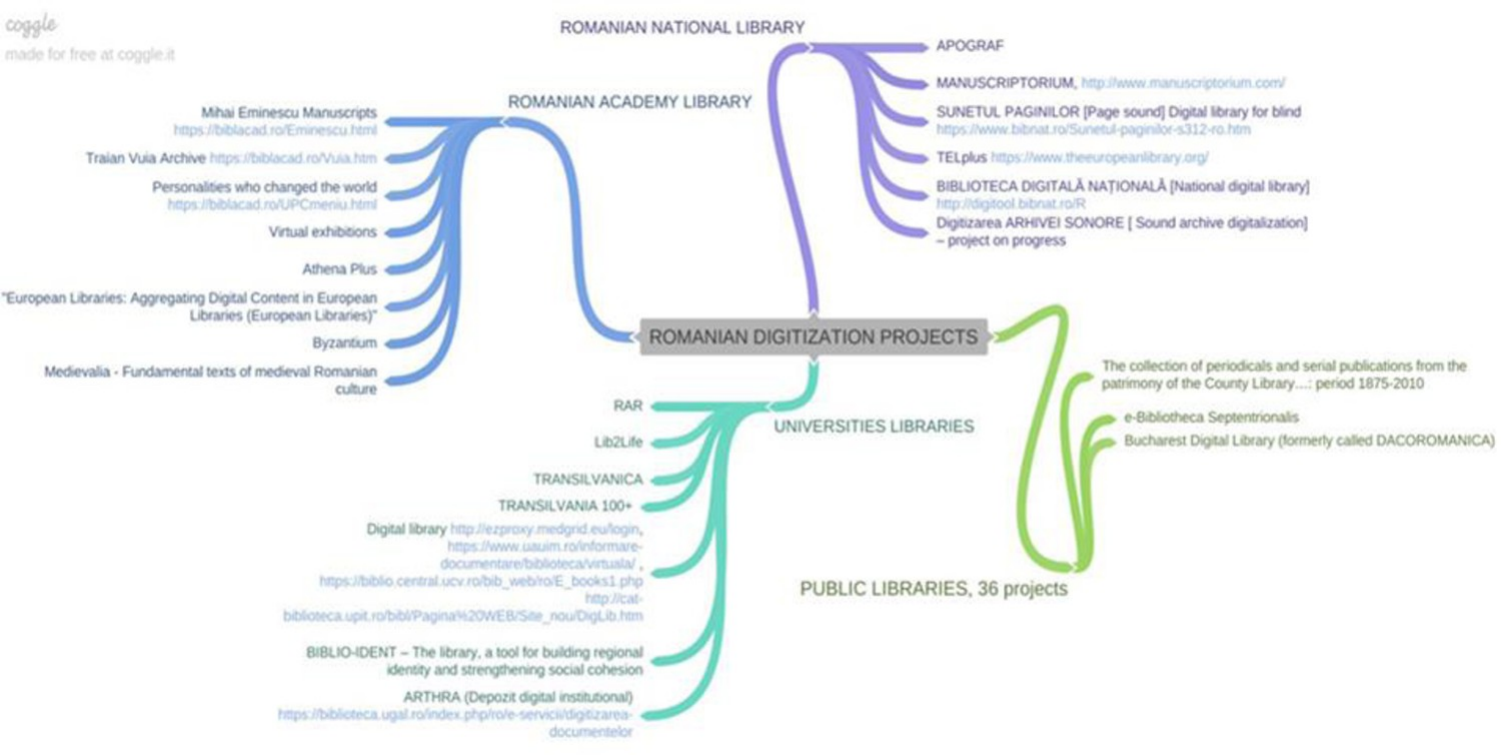
Romanian digitisation projects of own collections.

Initiatives and digitisation projects have been identified at the level of libraries or other cultural institutions in the country (Fig 6), such as the NLR, the “Carol I” Central University Library in Bucharest (about 20,000 pages–rare books, manuscripts, serial publications, current monographs), the Romanian Academy Library (more than 36,000 facsimilia of “Eminescu Manuscripts”, in collaboration with the Ministry of Culture and Cults and cIMeC (Institute of Cultural Memory), as well as the “Traian Vuia” archive), the Metropolitan Library in Bucharest, the “Panait Istrati” County Library in Braila (Romanian literature bibliography according to school curriculum), “Gh. Asachi” County Library in Iasi (over 2594 pages and 950 images), and the “Octavian Goga” County Library in Cluj-Napoca. The Braşov County Library together with the “Casa Mureşenilor” Foundation digitise the “Gazeta de Transylvania” publication and one can speak of ongoing activity in both these institutions and other institutions in the system.

Many libraries present, on their own websites, documents or digital collections, not digital libraries. A digital library is an information service that provides users’ access to digitised documents through a digital library application and a relational database. The quality of services provided by a digital library refers to the degree of accessibility, navigation facilities, collection consistency, content quality, quality of digital objects, metadata quality, use level, and compliance with Copyright Law.

Digital library applications used in Romanian libraries are Digitool (the NLR, the Metropolitan Library in Bucharest), Greenstone (Cluj County Library, Neamţ County Library, Maramureş County Library, Galati County Library), D-Space (Cluj Central University Library, Library of the Danube University in Galati), and Toread (Braila County Library, the University Library in Craiova) [20].

Below is the presentation of two digital Romanian libraries considered as examples of good practices in the field.

The National Digital Library [21] consists of digital collections created by digitizing documents organized by themes or after events. Currently (February 2022, the date of completion of this study), it comprises over 14,500 documents (To note that a digital document is statistically a single digital unit regardless of size or number of pages, therefore, a book is a single digital object just like a photo. To estimate the size of a digital library, the number of documents contained is not the most relevant indicator–the amount of existing digital information should be assessed. A real and relevant assessment of a digital library can be done by qualitative indicators and use) organized in the following collections:

- 2590 documents in the historical Archive made up of the historical document Fund from the 15^th^– 19^th^ c., Kogălniceanu Fund, Saint Georges Fund, Brătianu Fund, and 20^th^ c. Fund;
- 1046 resources presented in virtual Exhibitions about Al. I. Cuza, the First Romanian astronomic expedition, total Sun Eclipse 1893, Romanian army campaigns, Dragomir Hurmuzescu, Romanian feminine personalities, etc.
- 149 incunabula;
- 720 old Romanian periodicals;
- 740 old Romanian and bibliophile resources;
- 2917 Romanian and European photographs, Romanian illustrated postcards, postcards illustrated from Bessarabia and Bucovina, photo albums, etc.;
- 1010 manuscripts including Arab and Greek manuscripts, as well as documents from the Batthyaneum library collections;
- 1210 foreign old books;
- 384 cartographic material (atlases, old maps, etc.);
- 1542 musical scores;
- 102 ex-libris, 107 Japanese stamps, 33 Romanian drawings, 21 engravings, 5 art albums, etc.;
- 790 civil documents such as correspondence, old newspapers items, and military documents such as reports, sketches of the situation of military units on the battlefield at different times in history, brochures, informative notes, confidential notes, etc., presented in the collection Romania in the First World War;
- Other thematic collections constituted as a result of a project for an assessment or request from a beneficiary.

From the collections of the National Digital Library administered by the National Library, 4686 digital objects are also exposed in European: historical archive (1878), incunables (153), Romanian old books (316), old books (876), manuscripts (448), musical scores (146), Japanese stamps (107), photos (434), drawings (51), and ex-libris (90).

Romania’s digital library is an example of good practice because it is organized in its entirety according to international norms and standards accepted for digital collections and libraries, for several reasons. Bit presents collections of different types of documents so that examples of specific digitisation, processing, publication, and accessibility can be analysed. Technically and “librarianshipically”, it allows integration and relationship with similar European structures such as Europeana, Manuscriptorium, TEL–The European Library, and its legal and administrative functioning framework is well-defined by the Library Law, by ministerial orders.

The Bucharest Digital Library [22] is a project supported by the Bucharest General City Hall through an investment of about 3,000,000 euros, consisting of performing equipment that constitutes the scanning infrastructure (10 professional scanners A2-A0), processing (workstations), professional software (processing and OCR [Optical Character Recognition] -isation), archiving, as well as Digitool professional digital library application, a DAMS (Digital Assets Management System) application–which has implemented content standards, metadata, display, and interoperability (Z39.50, OAI-PMH). The Bucharest Digital Library project was launched in 1999. Currently, the digital library of Bucharest provides access to a fund of over 60,000 documents amounting to about 10,000,000 pages, several hundred images and several digitized sound resources. In the heritage of the Bucharest Digital Library, a documentary fund dedicated to local community memory was established, including reference works concerning the history of the capital, such as N. Iorga (Istoria Bucureştilor, Bucureştii de acum un veac, Conducător istoric 89 la bisericile din Bucureşti şi împrejurimi), G. Ionescu-Gion (Istoria Bucureştilor), D. Pappazoglu (Istoria fondării oraşului Bucureşti), I. Licherdopol (Bucureşti), C. C. Giurgescu (Istoria Bucureştilor), Frédérique Damé (Bucarest en 1906), H. Stahl (Bucureştii ce se duc), G. Potra (Documente privitoare la istoria oraşului Bucureşti, Din Bucureştii de altădată), D. Caselli (Cum au fost Bucureştii odinioară), A. Bacalbaşa (Bucureştii de altădată), P. Morand (Bucarest). They also included in this project collections and documents from the Romanian Academy Libraries, from “Nicolae Iorga” Institute of History, and from the National Museum of Romanian Literature.

The Bucharest Digital Library is an example of good practice because it has since been a project undertaken by the patronage development institution and authority and has benefited from the necessary investments; because it is organized in its entirety according to international norms and standards accepted for digital collections and libraries; because it has managed to cooperate with other document-owned institutions and to integrate into a unitary document structure from disparate collections; because it has developed a Documentary Charter to organize digitisation and it has managed a good promotion of its information service in the public area.

### Involvement of Romanian libraries in European digitisation projects

Representative libraries in Romania are involved in European projects. Participation in such projects is, for the Romanian institutions, an opportunity to integrate into the European vocational community and to make Romanian cultural heritage and its interference with European and global cultural heritage internationally known [23]. Below are three such projects considered representative and models of good practices.

EDL Local–Europeana Local [24] is a European project funded by the European Commission through the eContentplus programme–Best Practice Networks–Networks of Good Practices. Thirty-two partners from 27 European countries have been involved in its implementation. EDL Local is based on the same objectives and principles as the EUROPEANA project being a complementary project. The project aims to support good practices of digitisation activities and provide access to local or regional content through the European Digital Library. The project was carried out during 2008–2011. Romania was involved in this project through county libraries, museums and archives, and the results can be considered a remarkable example of good practice in the field of digitisation, collaborative, and project work.

The declared objectives of the project [24] are: support for European regions to implement Europeana infrastructure and standards; activities to stimulate aggregation agreements in related areas (e.g., with national libraries or cultural portals); promoting digitisation at local or regional level; promoting and facilitating participation in Europeana of other local/regional digital content owners.

The Romanian partners were the “Octavian Goga” County Library in Cluj-Napoca (coordinator partner); the National Archives in Cluj-Napoca; the “Alexandru and Aristia Aman” in Dolj County; “G. T. Kirileanu” Library of Neamţ County; the “Gh. Asachi” County Library in Iasi; the “Ovid Densușianu” County Library in Hunedoara; the “Panait Istrati” County Library in Braila; the “V. A. Urechia” County Library in Galati; the Astra Sibiu County Library; the Timiş County Library; the County Centre for the Promotion and Conservation of Traditional Culture in Cluj-Napoca; the Museum of Dacian and Roman Civilizations in Deva.

Digitized collections integrated into Europeana consist in manuscripts, books from local history collections, monographs, old periodicals, images, audio documents, video documents. Twenty-six collections of about 13,314 digital documents were estimated. The collections continued to develop and diversify and, after the end of the project, other libraries joined this approach.

Examples of collections and documents in Europeana as a result of the project [25] are: Images from the old Cluj; Archive documents from the collections of the County Library in Cluj-Napoca; Documents from the special collections of the “V. A. Urechia” County Library in Galati (diplomas, lithographs, photos, stamps); Local history in books and periodicals from the collections of the Neamţ County Library; Banat writers from the collections of the Timiş County Library; Images from yesterday and today’s Craiova from the collections of the Dolj County Library.

Once the project was completed, durability was accomplished at institutional level through the library’s own budget and, thus, ensured permanent access to the resources included and enrichment of content by adding new resources. Subsequent to the local EDL project, the concerns related to the addition of Romanian digital content to Europeana were continued through other initiatives (the LoCloud Project) and through the voluntary contribution supported by a series of libraries.

cIMeC–Cultural Memory Institute [26], by participating in European projects and aiming at contributing to the development of the European Portal through the involvement in the development of the field at national level, has helped to crystallize the achievement initiatives of the Digital Library of Romania. The institution concerned was involved in the projects European Digital Library Network (EDL-Net), ATHENA (financed by the project e-ContentPlus, 2008–2011) and EUROPEANA 1 (funded by e-ContentPlus, 2009–2010) [27].

The NLR has been involved in international projects aimed at creating relevant digital content for European cultural memory. The contribution of the National Library consists in the representation and promotion of national cultural heritage and its integration into the European and world heritage. Since 2007, the institution has been involved in the following projects:

- TELplus [26]–The European Library (TEL). The project was funded within the eContentplus program and was coordinated by the National Library of Estonia. It started in October 2007 and lasted 27 months. One of the main objectives of the project was to add collections of National Libraries from Bulgaria and Romania to the European collective catalogue and to support these two institutions to get full membership in TEL, thus achieving a single point of access to the resources of Europe’s national libraries. The activities corresponding to this objective have been found in the activities of the sixth set of the structure and planning of the project, Work Package (WP) 6: Increasing the number of members of the European Library portal by adding national libraries from Bulgaria and Romania. The leader of this WP was the National Library of the Netherlands–The European Library Office. The actual activities of the NLR consisted in [28] information exchange between TEL Office and the NLR–questionnaires, teleconferences–to determine how to integrate collections in the portal; inclusion of the collections of the NLR in the portal as browse-only; translation of the portal interface www.theeuropeanlibrary.org into Romanian; integration of collections through OAI-PMH; purchase of a new library system; data conversion; testing; marketing plan; participation of library representatives in working group meetings (The European Library Technical Working Group); promoting the European Library project at numerous events attended by the NLR; articles, studies and presentations; installation of the minisearch box in the direct search library website in the www.theeuropeanlibrary.org portal.

Since January 2009, the NLR is a full member of the European Library and, as such, contributes to the promotion of Romanian documentary heritage (in the form of bibliographic and full-text records) at European and international level.

- ENRICH–European Networking Resources and Information Concerning Cultural Heritage [29] / Manuscriptorium [29], as presented in the previous chapter, is a project developed under the eContent+ programme. The aim of the project is to ensure access to digital images of old documents from different European cultural institutions by creating a virtual research environment dedicated primarily to the study of manuscripts and of incunabula, old and rare printed books and other historical documents. Access is available through the Manuscriptorium digital library portal. The NLR is a partner in the Manuscriptorium project since April 2008 and it has contributed 109 Romanian books from the 16^th^-18^th^ c., with a special cultural, historical and artistic value, as well as with 358 precious manuscripts from the Batthyaneum branch collections. These materials have a special historical and artistic value. Most of these heritage works are religious, but there are also books of law or history.

Since Manuscriptorium integrates with the European library portal (The European Library) and with the European digital library (Europeana), the documents in the collections of the NLR are also found in the latter.

- REDISCOVER (Reunion of Dispersed Content: Virtual Evaluation and Reconstruction [30]) is a project coordinated by the National Library of the Czech Republic carried out under the European Union Culture Programme 2007–2010. The project partners were the National Library of Poland, the National Library of Lithuania and the NLR. The project is presented in the previous chapter of this study.
- Balkan Itineraries. “Digital collections online” is a project in partnership with “St. Klimet Ohridski” University Library in Sofia, Bulgaria, with the “Svetozar Markovic” University Library in Belgrade, Serbia, and l’Agence Universitaire de la Francophonie (AUF), Bureau Europe Centrale et Orientale (BECO). The project aimed at achieving a documentary digital francophone corpus of rare and precious books from these Balkan libraries and the publication of the collection on the Internet.

In order to point out the incidence of digitization in Romanian libraries, the ongoing projects developed by Romanian libraries were examined from which the most relevant ones for the Romanian area (the relevance criterion relies on the visibility on the European library portal and on the professionalism with which they were developed) were selected for presentation as good practices.

## Discussions. Evaluation of results

The needs of the current public regarding libraries are directed to hybrid collections (print and electronic formats), and the public in Romania becomes demanding as far as the online services provided by the library are concerned, so it is necessary to accelerate the rate of digitisation of collections to avoid much larger gaps between EU countries; in addition, the services provided by libraries in the electronic information area are in competition with other service providers (publishers, universities, etc.).

Developing digital content are obviously a major priority objective of libraries. Libraries are present in the digital environment and they engaged in digital content in the desire to extend the number and typology of specific users, the need for diversification of the products and services provided in accordance with the needs of users, the desire for the most diversified use of the heritage, for reasons of protection by digitizing documents and collections.

Over the past 15 years (2007–2022, the period analysed in this study), there have been numerous digitisation initiatives and projects. The success or failure of a program or project of digitisation of the patrimonial collections in libraries, including the project of the Digital Library of Romania, depend on the understanding of the functional realities of Romanian libraries in which each type of library has a specific legal and administrative framework of operation, functions and specific attributions, determinations and limitations in decisions and cooperation. Assuming, by the authorities, with responsibility a coherent strategy for digitisation projects to produce perennial effects is also an elementary condition. The experience of Romanian libraries shows that digital content development initiatives have not always been successful because of the absence or insufficiency of the sustainability component.

The analysis and interpretation of the data collected on the Romanian context clearly shows that important steps have been taken to digitize the national documentary heritage, even if these steps have not been financially supported in accordance with the Action plan developed on the basis of public policy for the digitisation of cultural resources approved by the Romanian Government in 2008. Because of the lack of funds, many projects or ongoing digitisation activities have been restricted or stopped, and already digitized funds are not available to the public because they lack the software for processing and integration into collections and publication sites.

The absence of a national funding program that include at least representative libraries in the National Library System resulted in chaos and waste in digitisation activities: each library digitizes what its managers want and they want to do it without a subordination to a national strategy and without trying to avoid doubling: this explains the existence of the same digitized collections in several libraries (for example, the case of periodicals or school bibliography). Effective digital storage and archiving solutions are not considered and only local storage (more or less empirical) is achieved. The processing (cataloguing and processing of digital objects) is not carried out in all cases according to metadata, standards and international norms; thus, when the possibility of integrating into digital databases and libraries, a new processing is needed.

The E-Culturalia project has tried to harmonize digitisation practices in Romanian cultural institutions and provide examples and good practices needed for the libraries in the Romanian National System of Libraries. The coordinator of the Digital Library of Romania should be the NLR that has attributions in this respect established by the Library Law and by the secondary legislation elaborated by the Ministry of Culture. Relevant in this respect are the thematic studies and analyses carried out at the national library system level; inventory of projects and documents digitized in libraries, involvement in the development of the digitisation guide, activities already presented in this study. But the greatest impediment can be considered insufficient or lack of collaboration of libraries in the system. If Romanian libraries, particularly large university and county libraries, do not formally recognize the coordinating and methodological role of the NLR, there will be no visible progress in achieving the necessary National Library System projects such as the Digital Library of Romania or the Shared National Catalogue and there will be individual or collective projects in just a few institutions, without real national representation, without real responsibility at central authorities level and without real long-term sustainability. An institutional project may be a component of a project of the national library system, but it cannot substitute it.

Good practices for digitizing the written cultural heritage from libraries mean numerous digital contents valuable from a cultural, scientific, and heritage point of view. They are carried out, processed and published in compliance with the standards and technical, ethical, legislative, administrative norms. Such programs and projects will be the core of the National Digital Legal Repository.

## Conclusions

Digitization projects are important: they represent the best method of visibility (understood as information accessibility) and of preserving the documentary fund. Libraries have been aware of this need and, in Romania, many local projects have been materialized (S1 Appendix). The experience of Romanian libraries for the digitisation of heritage shows the opportunities and the limits imposed by the inconsistency of common application and consistent digitization policies.

The originality of this study is that the authors monitored the process of carrying out a national and coherent digitisation policy at the level of Romania, started in 2007, under the coordination of the National Library. The initial feasibility study has led to the emergence of the CASIDRO Information product-database in continuous update regarding the activity of Romanian libraries, an active, sustainable, reference referential. From the large volume of information collected and analyzed, the following aspects resulted in synthesis: the analysis of the digitisation process, as a public policy of the libraries in Romania, the exposure of the general and specific principles of setting up the national digital deposit, for public accessibility in the online environment and to protect the Romanian documentary heritage. This study contributes to the formation of an overview of libraries as public organizations. The descriptive case study rigorously presents the process of setting up and implementing the constitution and implementation policies of the digitization of the documentary heritage from the perspective of the Romanian libraries as public institutions, focusing on the large libraries, which have heritage funds–the National Library, University and county libraries–as actors capable of supporting such an approach from the perspective of logistics and staff. By monitoring the dynamics of the digitisation process in Romanian libraries, this study creates the opportunity to share experience with other researchers, signalling the advantages / limits to be taken into account or avoided by other libraries in the world.

There are no previous such studies in Romania, so this study is the first, original and important one because it theorizes a process. It completes the lack of literature on digitisation projects in Romania. Great efforts have been made, there is an important digitized fund, and awareness should be raised of decision-makers that digitisation of heritage is the key method of sustainability of values through their digital preservation.

Collective efforts may lead to the protection of national heritage. The experience of the last two years (the COVID-19 pandemic period) has demonstrated the need for digital information and for intensifying joint digitisation projects for collections and sustainability for future generations, to create new opportunities for science and education.

Other national experiences will be subjected to research.

## Supporting information

S1 Appendix Update of digitization projects—Romanian libraries. (DOCX)
