# Supplementary material for: Visibility of documentary heritage through digitisation projects in Romanian libraries
Source: PLoS One. 2023 Jan 23;18(1):e0280671. doi: 10.1371/journal.pone.0280671 (PMC9870124; doi:10.1371/journal.pone.0280671)
Supplement: S1 Appendix — (DOCX) [file pone.0280671.s001.docx]

**Appendix A. Update of Digitization projects — Romanian libraries**

**Material made with the support of Cristina DOVÂNCĂ, PhD, BCU „Carol I” Bucharest, References service**

| **No. crt.** | **Project name** | **Description/ Site** | **Collaboration** |
| --- | --- | --- | --- |
| **NATIONAL LIBRARY OF ROMANIA** | | | |
| 1. | Apograf | In 2003-2004 the digital scanning of 100,000 pages of manuscripts and prints of high bibliophilic and documentary value was carried out from the collections of the Batthyaneum Filial Library.  In 2020, the collection of Exlibris of the Batthyaneum Library’s documentary heritage was scanned and processed, which can be viewed in the National Digital Library. | It is part of the national project The National Digital Library of Old Manuscripts and Books (started by the Ministry of Culture and Religious Affairs through cIMeC) |
| 2. | Manuscriptorium | <http://www.manuscriptorium.com/>  (December 2007-November 2009) — the portal of European manuscripts, the objective of which is to ensure unhindered, distributed access to European documentary heritage specialised in manuscripts, incunabulas, old and rare books, and other historical documents.  He managed to integrate collections from over 46 representative institutions in Europe, especially from National Library, 85 %. | Funded by the eContentPlus program, coordinated by the Czech National Library |
| 3. | The Sound of the Pages | <https://www.bibnat.ro/Sunetul-paginilor-s312-ro.htm>  Digital Library for the Blind | Financially supported by Orange Foundation through the “World through Color and Sound” Program |
| 4. | TELplus | <https://www.theeuropeanlibrary.org/> | Funded by the EU’s eContentplus program for a period of 27 months (October 1, 2007 - December 31, 2009) |
| 5. | National Digital Library | <http://digitool.bibnat.ro/R>  Purpose: onserving and protecting the national cultural heritage existing in the libraries of the National System of Libraries as well as promoting collections and widening access to information. Documents created only in electronic format (online books and magazines, electronic resources from websites, databases, etc.) will be included in the National Digital Library.  In 2020, 720 old and rare documents were published in the National Digital Library, amounting to 57.965 images, published in the *National Digital Library*.  In order to better promote digital objects uploaded in the Digital Library, a section has been created on the website of the National Library where the latest scanned and published documents can be viewed: <https://www.bibnat.ro/Ultimele-documente-digitizate-s392-ro.htm>. | Part of the European Digital Library |
| 6. | Digitisation of Sonore Arhive  (project ongoing) | BibNat’s sound archive contains 7153 ebonite discs at 78r/min., intended for the patephone, discs released at the end of the 19th century and a number of 20825 vinyl discs at speeds 33r/min and 45r/min.  Instructions and handling rules were drafted in 2020 in order to achieve the digitisation process and costs were estimated. | A financing solution is being sought |
| **LIBRARY OF THE ROMANIAN ACADEMY** | | | |
| 7. | Mihai Eminescu Manuscripts | <https://biblacad.ro/Eminescu.html>  46 volumes, approximately 14,000 tabs | In collaboration with the Ministry of Culture and Religious Affairs and CIMeC |
| 8. | Traian Vuia Archives | <https://biblacad.ro/Vuia.htm>  It contains the facsimiles of manuscripts and plane plans invented by Traian Vuia. |  |
| 9. | Personalities who have changed the world | <https://biblacad.ro/UPCmeniu.html>  The project started in October 2010 with the digitisation of a valuable selection of manuscripts and rare works, owned by the Library of the Romanian Academy, and this section will be completed in time with authentic world cultural landmarks. | In collaboration with UPC |
| 10. | Virtual exhibitions | <https://biblacad.ro/expoVirtuale.html>  Al. Al. Ioan Cuza: 200 years since birth  How to create modern Romania  From the world of Romanian press: 191 years  Romania-France: cultural itineraries  Constantin Brâncoveanu  Historical seals  Orghidan Collections: Transylvanian coins from the Constantin Orghidan collection: photo gallery and exhibition film  Stefan Luchian  Vasilica Chifu  Antim Ivireanu  Argeș Monastery Church  Ferdinand I The Entity  Interference: Marius CRISTEA painting exhibition |  |
| 11. | Athena Plus | 2013-2015  The main objective of the AthenaPlus project **is** to contribute more than 3.6 million metadata records to *Europeana*, both from the public and private sectors, focusing mainly on museum content, with key cultural actors (ministers and responsible government agencies, libraries, archives, leaders). | 40 partners from 21 European countries |
| 12. | European Libraries: Aggregation of digital content in Europe’s libraries (European Libraries) | 2011-2012  The Library of the Romanian Academy is the content provider of this project (for Europeana). | Coordinated by KB (National Library of the Netherlands) and supported by three long-standing European bodies (CENL, LIBER, CERL). A large consortium of partners, from 11 countries, which are national or research libraries |
| 13. | Byzantion | From the rich manuscripts (over 10,000 odds) of the Romanian Academy Library were selected manuscripts ornate and miniatures, Greek (15), Romanian (108) and Slavic (27), some exceptional by artistic conception and achievement, including some fundamental iconographic themes. |  |
| 14. | Medievalia — Fundamental Texts of Medieval Romanian Culture | https://medievalia.com.ro/ | Part of the Program PA16/RO12 “Preservation and Revitalisation of Cultural and Natural Heritage” — Small Grant Scheme, financed by the EEA Financial Mechanism 2009-2014 with the Ministry of Culture as Project Operator |
| **CENTRAL UNIVERSITIES LIBRARIES** | | | |
| **CENTRAL UNIVERSITY LIBRARY “CAROL I”, BUCHAREST** | | | |
| 15. | Restitutio | <http://restitutio.bcub.ro/>  It contains about 20,000 pages — old and rare books, manuscripts, magazines and other serial publications, current Romanian and foreign monographs, iconographic resources, cartographic resources and audio-video resources. |  |
| 16. | RAR — The recovery of Anglistics in Romania | <http://www.recuperareaanglisticii.ro/catalog-carti.html>  The project consists of protecting, capitalising and promoting the cultural heritage written in the field of Romanian anglistics by identifying a documentary segment of about 250 volumes and digitising them. | Initiated by the University of Bucharest and realised in partnership with the Library of the Romanian Academy |
| 17. | Lib2Life — Revitalisation of libraries and cultural heritage through advanced technologies | <http://lib2life.ro/> | Consortium of 4 BCUs, Polytechnic University and National Institute for Research and Development in Informatics |
| **CENTRAL UNIVERSITY LIBRARY “LUCIAN BLAGA”, CLUJ-NAPOCA** | | | |
| 18. | Transylvanian | <https://documente.bcucluj.ro/>  Documents digitised in BCU “Lucian Blaga” Cluj-Napoca, books, manuscripts and periodicals whose author is of Transylvanian origin, were written or published on the territory of Transylvania. |  |
| 19. | Transylvanian School | <https://documente.bcucluj.ro/scoala/#gsc.tab=0> |  |
| 20. | Transylvania 100+ | <http://www.transilvania100plus.ro/index> |  |
| 21. | Digital Library | <https://dspace.bcucluj.ro/> |  |
| **CENTRAL UNIVERSITY LIBRARY“MIHAI EMINESCU” IASI** | | | |
| 22. | Digital Library | <http://dspace.bcu-iasi.ro/> |  |
| **CENTRAL UNIVERSITY LIBRARY“EUGEN TODORAN” TIMISOARA** | | | |
| 23. | Digital Library | <https://bcut.ro/biblioteca-digitala/> — inthe course of editing |  |
| 24. | Biblio-IDENT.  “Library, tool to build regional identity and strengthen social cohesion — Biblio-Ident” | <http://bcut.ro/biblioident/>  The aim is to create a database of heritage books (scaning, indexing and translating the title page and the contents of the 500 books selected for the “Banatica Fund” in four languages: English, Serbian, Hungarian and German), as well as scanning the content of 100 books in full. The results anticipated within the project will be translated into a common database, including heritage books about the Banatica region, called “*Banatica Fund*”. | Cross-border project Romania-Serbia |
| **UNIVERSITIES LIBRARIES** | | | |
| **LIBRARY OF THE UNIVERSITY OF MEDICINE AND PHARMACY “CAROL DAVILA”, BUCHAREST** | | | |
| 25. | Virtual Library U.M.F. Carol Davila | <http://ezproxy.medgrid.eu/login> |  |
| **LIBRARY OF “ION MINCU” UNIVERSITY OF ARCHITECTURE AND URBANISM, BUCHAREST** | | | |
| 26. | Virtual Library “Ion Mincu” | <https://www.uauim.ro/informare-documentare/biblioteca/virtuala/> |  |
| **UNIVERSITY OF CRAIOVA LIBRARY** | | | |
| 27. | Digital Library | <https://biblio.central.ucv.ro/bib_web/ro/E_books1.php> |  |
| **LIBRARY OF “THE LOWER DANUBE” UNIVERSITY OF GALATI** | | | |
| 28. | Arthra (Dinstitutional digital repository) | <https://biblioteca.ugal.ro/index.php/ro/e-servicii/digitizarea-documentelor>  Operational since 2011, it includes documents that have been subject to digitisation (printed publications) and “digital born” documents. |  |
| **UNIVERSITY LIBRARY OF PITESTI** | | | |
| 29. | Digital Library | <http://cat-biblioteca.upit.ro/bibl/Pagina%20WEB/Site_nou/DigLib.htm> |  |
| **LIBRARY OF THE UNIVERSITY OF MEDICINE, PHARMACY, SCIENCE AND TECHNOLOGY “GEORGE EMIL PALADE”, TARGU-MURES** | | | |
| 30. | Digital Library | <http://dspace.umfst.ro/jspui/?locale=ro> |  |
| **PUBLIC LIBRAIRES** | | | |
| **BUCHAREST METROPOLITAN LIBRARY** | | | |
| 31. | Digital Library of Bucharest (formerly called Dacoromanica) | <http://digibuc.ro/>  Implemented by the Metropolitan Library since 2009.  Provides access to digitised documents such as text, image, audio or video, true digital copies of documents belonging to both B.M.B. heritage and other institutions holding heritage funds (Romanian Academy Library, Institute of History “Nicolae Iorga”, Museum of Romanian Literature). |  |
| **“ALEXANDRU D. XENOPOL” COUNTY LIBRARY ARAD** | | | |
| 32. | Digital Library | <https://digital.bibliotecaarad.ro/>  Digital Library dedicated to local culture and history. |  |
| **“DINICU GOLESCU” COUNTY LIBRARY ARGEȘ** | | | |
| 33. | Encyclopedia of Arges and Muscel | <https://www.bjarges.ro/biblioteca/profesional/proiecte/enciclopedia-argesului-si-muscelului/> |  |
| **COUNTY LIBRARY “PETRE DULFU” LARGE BATHROOM** | | | |
| 34. | e-Bibliotheca Septentrionalis | <https://ebibliothecaseptentrionalis.wordpress.com/2021/07/22/a-fost-digitizata-toata-colectia-calendarului-romanesc/> |  |
| **“GEORGE COŞBUC” COUNTY LIBRARY BISTRIȚA-NĂSĂUD** | | | |
| 35. | Old book | <https://www.bjbn.ro/carteveche/> |  |
| 36. | Books from the library of George Coșbuc | <https://www.bjbn.ro/carti/> |  |
| 37. | Manuscripts | [**https://www.bjbn.ro/manuscriselovinescu/**](https://www.bjbn.ro/manuscriselovinescu/) (Eugen Lovinescu)  [**https://www.bjbn.ro/manuscrisemanolescu/**](https://www.bjbn.ro/manuscrisemanolescu/) (N. Manolescu)  <https://www.bjbn.ro/manuscrisepetru/> (Peter Poant) |  |
| 38. | Publications, Editorial projects | <https://www.bjbn.ro/publicatii/> |  |
| **“GEORGE BARIŢIU” COUNTY LIBRARY BRASOV** | | | |
| 39. | Digital Library | <https://www.bjbv.ro/scan/scan.php>  Contains:  “Gazeta DE TRANSILVANIA”  (<https://www.bjbv.ro/scan/gt1848/gt-1848.html>)  “Sacelen plaiuri”  (<https://www.bjbv.ro/scan/plai/plai.html>)  ‘OCTOICH’ (<https://www.bjbv.ro/scan/octoich/octoich.html>)  The “Glass of YOUTH”  (<https://www.bjbv.ro/scan/glas/glas.html>)  “Glas of Ardeal”  (<https://www.bjbv.ro/scan/glas/glas.html>) | In collaboration with the Foundation “Casa Mureşenilor” (Gazeta de Transilvania) |
| **“PANAIT ISTRATI” COUNTY LIBRARY BRĂILA** | | | |
| 40. | School Digital Library/Literature English | <https://www.bjbraila.ro/literatura-romana/> |  |
| 41. | Digitisation of local media | <https://toread.bjbraila.ro/opac/authority/108563;jsessionid=84E57BBF5148A498294D48ACC337201D> |  |
| **“OCTAVIAN GOGA” COUNTY LIBRARY, CLUJ-NAPOCA** | | | |
| 42. | LoCloud Project | <https://www.bjc.ro/new/index.php?contributia-romaneasca-la-biblioteca-digitala-europeana/> |  |
| **“IOAN N. ROMAN” COUNTY LIBRARY CONSTANTA** | | | |
| 43. | Digitisation of BJConstantance | <http://digitizare.biblioteca.ct.ro/>  The digitisation project started in 2003 and targeted the collection of old periodicals in Dobrogea.  The current collection contains: books, iconography, manuscripts, periodicals, biblion magazine, etc. in digital format. |  |
| “BOD PETER” COUNTY LIBRARY COVASNA | | | |
| 44. | Digitisation | <http://www.kmkt.ro/digitalizalas>  It contains the three year series edited at St. Gheorghe of the *newspaper* Nemere (1877-1878-1879). The material came from the collection of the Szekler National Museum, was scanned, transformed from image into textual document, it was compiled a repertoire (description of articles, index and keywords), and the digitised material was published on the Internet, with professional assistance from the National Library of Hungary, within the program of drafting bibliographies *Humanus*, to which the library is associated. |  |
| **“ALEXANDRU AND ARISTIA AMAN” COUNTY LIBRARY DOLJ** | | | |
| 45. | Digital Archive | <https://aman.ro/arhiva-digitala/> |  |
| **“KÁJONI JÁNOS” COUNTY LIBRARY HARGHITA** | | | |
| 46. | Memory of Szeklerland in digitised document | <https://biblioteca.judetulharghita.ro/ro/h/130/documente-digitizate>  The digitisation activity in the “Kájoni János” County Library started in 2009 as part of the digitisation project of the ***Szeklerland memory in digitised document.*** | In collaboration with the Hungarian National Library and the Haáz Rezső Museum Library in Odorheiu-Secuiesc. |
| **“OVID DENSUSIANU” COUNTY LIBRARY, HUNEDOARA** | | | |
| 47. | Hunedoara Digital Library — facilitating access to local digital documents | <http://www.bibliotecadeva.eu/bdh.html> |  |
| 48. | The Fund of Periodics and Serial Publications from the Property Library: period 1875-2010 | <http://www.bibliotecadeva.eu/periodice/periodice.html> |  |
| **“I.A. BASSARABESCU” COUNTY LIBRARY GIURGIU** | | | |
| 49. | Digital Library | <https://www.bjgiurgiu.ro/diverse-carti/> |  |
| **MURES COUNTY LIBRARY** | | | |
| 50. | Mures Digital Library | <https://bjmures.ro/bd/index.php>  There are 196 digital documents, ordered after entry date |  |
| **COUNTY LIBRARY “G. T. KIRILEANU” NEAMT** | | | |
| 51. | Virtual Library | <https://bibgtkneamt.ebibliophil.ro/crw> |  |
| **“NICOLAE IORGA” COUNTY LIBRARY PRAHOVA** | | | |
| 52. | Digital Library | <https://biblioteca-judeteana-nicolae-iorga-ph.webnode.ro/biblioteca-digitala/> |  |
|  | School Library | <https://biblioteca-judeteana-nicolae-iorga-ph.webnode.ro/de-ale-scolii2/> |  |
| **“ASTRA” COUNTY LIBRARY SIBIU** | | | |
| *53.* | The Digital Library of ASTRA County Library Sibiu | <http://www.dspace.bjastrasibiu.ro/?locale=ro>  The digital library contains books, manuscripts, periodicals and iconographic materials from the collections of ASTRA County Library Sibiu. |  |
| **“SORIN TITEL” COUNTY LIBRARY TIMIS** | | | |
| 54. | Periodicals | <https://www.bjt.ro/periodice/> |  |
| 55. | Banat writers | <http://old.bjt.ro/bv/ScritoriBanateni/> |  |
| **“PANAIT CERNEA” COUNTY LIBRARY TULCEA** | | | |
| 56. | Local Virtual Library | <https://tulcealibrary.ro/portal/biblioteca-virtuala-locala/> |  |
| **“ANTIM IVIREANUL” COUNTY LIBRARY VÂLCEA** | | | |
| 57. | Collection “Country Library” | <https://www.bjai.ro/colectia-biblioteca-judeteana/>  It contains magazines (Book House, Cultural Vitrals, Adolescent...), books and digital documentaries. |  |
